# Minimally invasive surgery for removal of migrated intrauterine devices in a postmenopausal women

**DOI:** 10.1007/s00404-024-07438-w

**Published:** 2024-03-16

**Authors:** Ying Jin, Tenglan Wu, Jianmei Liao, Ying Liu, Xiaoqin Gan

**Affiliations:** Department of Obstetrics and Gynecology, Chengdu Women’s and Children’s Central Hospital, School of Medicine, University of Electronic Science and Technology of China, Riyue Avenue, Chengdu, Sichuan China

A 58-year-old woman was admitted to our hospital for the removal of intrauterine devices (IUD). She had an IUD insertion performed 25 years prior, but the specifics of the procedure were not documented. She was asymptomatic and had her last period 5 years ago. Ultrasonography showed an IUD at the juncture of the uterus's lower segment and the cervix. In addition, radiography revealed a circular IUD within the pelvic cavity and a T-shaped IUD in the left iliac fossa region (Fig. 1A). Pelvic CT scans exhibited a circular metallic shadow in the interspace between the bladder and the uterus (Fig. 1B), and a T-shaped shadow in the left iliac fossa (Fig. 1C).

She received hysteroscopy and laparoscopy under general anesthesia. The hysteroscopy did not locate any IUDs. However, laparoscopy revealed a circular metallic IUD located between the lower segment of the anterior uterine wall and the bladder (Fig. 1D). In addition, adhesions of the greater omentum and intestinal canal to the left anterior abdominal wall were observed, with a T-shaped IUD adhered to the area (Fig. 1E). Both migrated IUDs were successfully removed via laparoscopic surgery. Duration of the surgery was 40 min with approximately 5 ml of blood loss).

**Fig. 1:**
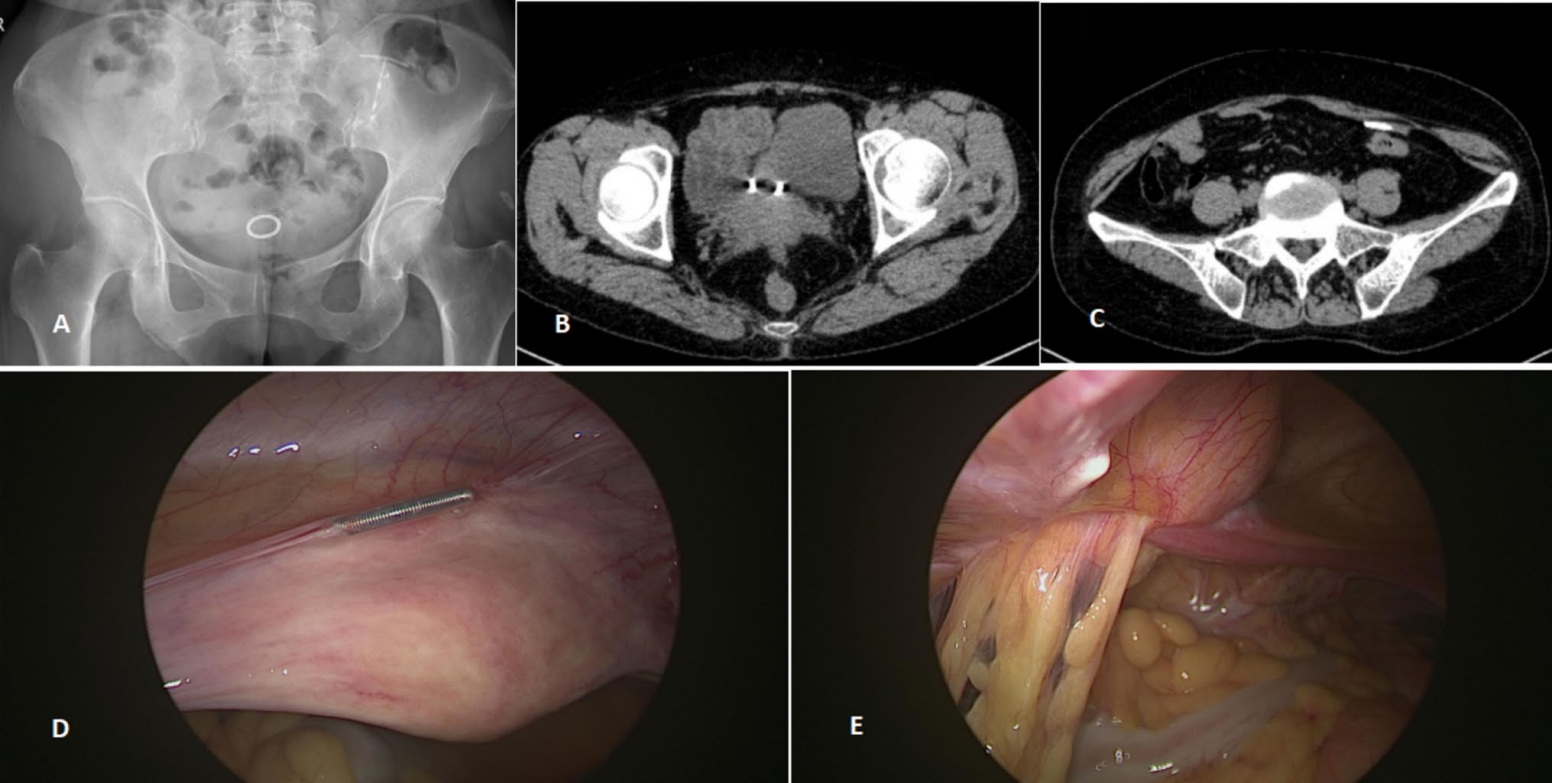
**A** X-ray showed an IUD in the pelvic cavity and another in the left iliac fossa. **B** and **C** Pelvic CT showed a circular IUD in the interspace between the bladder and the uterus and a T-shaped IUD in the left iliac fossa. **D** and **E** An IUD was seen between the anterior uterine wall and the bladder. Another was seen on the left anterior abdominal wall

Intrauterine devices are widely used all over the world, particularly in China during the 1980s to 1990s. A significant complication of IUD usage is uterine perforation, which can lead to potential IUD migration. The incidence of IUD migration varies between 0.1% and 0.9%. Typically, patients with migrated IUDs remain asymptomatic, as the IUDs tend to migrate through the uterus chronically without impacting other organs. Symptoms occur when migrated IUDs enter the abdominal cavity or perforate the intestine or other organs [1]. Ultrasonography is the preferred diagnostic tool for migrated IUDs, complemented by radiography [2]. Laparoscopic surgery is the recommended intervention for the removal of migrated IUDs from the peritoneal cavity [3].

## Data Availability

The data is available upon reasonable request to the corresponding author.
